# Elevated mature monocytes in bone marrow accompanied with a higher IPSS-R score predicts a poor prognosis in myelodysplastic syndromes

**DOI:** 10.1186/s12885-021-08303-8

**Published:** 2021-05-13

**Authors:** An Wu, Panpan Gao, Ningning Wu, Cong Shi, Zhenya Huang, Chunmeng Rong, Ye Sun, Lixia Sheng, Guifang Ouyang, Qitian Mu

**Affiliations:** 1 Department of Hematology, Ningbo First Hospital, Ningbo, Zhejiang PR China; 2 Institute of Hematology, Ningbo First Hospital, Ningbo, Zhejiang PR China; 3 Department of Hematology, Yuyao People’s Hospital of Zhejiang Province, Ningbo, Zhejiang PR China

**Keywords:** Mature monocyte in bone marrow, Myelodysplastic syndrome, IPSS-R, Prognosis

## Abstract

**Background:**

Myelodysplastic syndromes (MDS) is a group of heterogeneous myeloid clonal diseases originating from hematopoietic stem cells. Clinically, elevated mature monocyte in bone marrow is often observed, but its clinical value still remains unclear.

**Methods:**

We retrospectively analyzed a cohort of 216 MDS patients to explore the prognostic value of the percentage of mature monocyte in bone marrow (PMMBM). All patients were divided into elevated PMMBM group and the normal group by 6% PMMBM as the cut-off value.

**Results:**

Our results showed that PMMBM> 6% was associated with inferior overall survival (OS) (*P* = 0.026) along with higher-risk IPSS-R (*P* = 0.025) and higher frequency of IDH2 mutation (*P* = 0.007). Multivariate analyses showed that besides older age (> 60 years) for OS, gender (male) for OS, lower neutrophil count (< 0.8 ×  10^9^/L) for OS, higher bone marrow blast percentage (> 5%) for OS and LFS, poorer karyotype for OS, elevated PMMBM was also an independent adverse prognostic factor for OS in MDS (*P* < 0.0001) but not for LFS (*P* = 0.736).

**Conclusions:**

These findings indicate that increased PMMBM may assists *Revised International Prognostic Scoring System* (IPSS-R) to predict a poor outcome and provide a novel evaluation factor for MDS patients especially when their karyotype analyses fail.

## Background

Myelodysplastic syndromes (MDS), characterized by ineffective hematopoiesis, manifested by morphologic dysplasia in hematopoietic cells and peripheral cytopenia(s), is a group of heterogeneous myeloid clonal diseases originating from hematopoietic stem cells with a high risk of transforming to secondary acute myeloid leukemia (AML) [1]. The prognosis of MDS is extremely heterogeneous due to clinical and biological diversity. Herein, the International Prognostic Scoring System (IPSS) in 1997, the World Health Organization (WHO) Classification-Based Prognostic Scoring System (WPSS) in 2007, the MD Anderson Risk Model Score (MDAS) in 2008 and the Revised IPSS (IPSS-R) in 2012 were introduced to risk-stratify MDS patients [2–5]. Recently, lymphocyte-to-monocyte ratio and mutations such as TP53, SRSF2, IDH2 and ASXL1 were also demonstrated to predict the prognosis of MDS [6–10].

Before 2001, chronic myelomonocytic leukemia (CMML) was still categorized into MDS due to its MDS-like characteristics [11]. Unlike MDS, CMML is characterized by bone marrow (BM) dysplasia and persistent monocytosis, hence it is placed in a separate category of diseases [12]. Later, according to the 2016 revision of the WHO classification, CMML is subgrouped into 3-tiered blast-based categories: CMML-0 (peripheral blood (PB) < 2% and/or BM < 5%); CMML-1 (PB < 5%; BM < 10%) and CMML-2 (PB, 6 to 19%; BM, 10 to 19%) [13]. Under the prognostic model of IPSS-R, CMML-0 is in the very low and low-risk groups, CMML-1 in the low and intermediate groups, while most CMML-2 in the intermediate and high groups. Recently, Oligomonocytic CMML (OM-CMML) subtye (≥10% PB monocytes with absolute monocyte count (AMC) of 0.5–1 × 10^9^/L) is proposed. Previous literatures reported that some patients initially manifest MDS characteristics could progress eventually into CMML [14, 15], a part of which belong to OM-CMML.

The monocyte includes monoblast, promonocyte, immature monocyte, and mature monocyte [16], among which monoblast and promonocyte together with myeloblast are regarded as “blast” [12]. However, clinical value of mature monocyte in BM remains unclear for “true” MDS. Hence, 216 MDS patients in our cohort were retrospectively analyzed to measure the prognostic value of the percentage of mature monocyte in bone marrow (PMMBM). Our results suggested that increased PMMBM was an independent predictor for adverse outcome in MDS.

## Materials and methods

### Patients

Clinical and follow-up data of 216 patients were collected who were diagnosed of MDS in Ningbo First Hospital from 2009 to 2018. Diagnosis and classification of MDS and leukemic transformation were made according to the 2016 WHO classification [1]. Risk stratifications of MDS were made according to IPSS-R [5]. Cases with follow-up for less than 6 months or fulfill the diagnostic criteria of OM-CMML and CMML were excluded from the analysis. More than half of the patients received symptomatic and supportive treatment. Sixty-nine patients acquired further treatment, of whom 49 patients (22.7%) were treated with intensive chemotherapy, 16 patients (7.4%) with hemopoietic stem cell transplantation (HSCT) and 4 patients (1.9%), hypomethylating agents. The range of the percentage of mature monocyte in normal BM differs in different reports [17, 18], so we set a control group of 100 non-hematological malignancy cases to determine the PMMBM range (4.4% ± 1.8%; mean ± SD) (data not shown). Thus all 216 MDS patients were grouped into two groups basing on 6% PMMBM as the cut-off value for further analyses. Approval for the retrospective review of these records was obtained from the Ethics Committee of Ningbo First Hospital and was in accordance with the Declaration of Helsinki. Informed consent was obtained from all adult subjects or parents if subjects are under 18.

### Morphology analysis

The morphology of MDS myeloid cells were observed through Wright-Giemsa stained bone marrow smears. It was evaluated subjectively by light microscopy at low power (10 × objectives) for overall quality and distribution, and then was analyzed at high power (100 × oil objectives) for differential count including PMMBM count which was positively correlating with CD14 detected by flow cytometry (data not shown), with all cells in each containing field counted to maintain representative ratios of cell types [19]. All BM morphology findings were interpreted by two experienced and qualified clinical pathologists.

### Cytogenetic analysis

BM cells were collected and cultured in RPMI-1640 medium supplemented with 20% newborn calf serum for 24 h. R-banded metaphases were karyotyped according to the International System for Human Cytogenetic Nomenclature (2016) (ISCN2016) [20].

### Mutational analysis

Molecular analysis was performed as a part of the routine clinical work-up. Mutational analysis for 14 common genes of MDS including NRAS, DNMT3A, SF3B1, IDH1, IDH2, TET2, EZH2, JAK2, CBL, ETV6, TP53, SRSF2, ASXL1 and RUNX1 were performed with next generation sequencing.

### Statistical analysis

Statistical analyses were performed by SPSS 21.0. OS was calculated from the date of initial diagnosis of MDS to the date of death, last follow-up or acquiring allo HSCT. Leukemia-free survival (LFS) was determined from the date of diagnosis to the date of leukemia transformation, last follow-up or acquiring allo HSCT. OS and LFS were analyzed using the Kaplan-Meier method and compared using the log-rank test. Multivariable analyses were used by Cox proportional hazard regression model. Differences in the distribution of continuous variables between categories were analyzed by Mann-Whitney U and categorical variables by Chi-squared test. The receiver operating characteristic (ROC) curve was used to evaluate the diagnostic value of PB monocyte for MDS and the optimal cutoff value was 0.1 × 10^9^/L (data not shown). A *P* value of < 0.05 was considered statistically significant.

## Results

### Patients characteristics

A total of 216 patients of MDS including 99 females and 117 males were identified over a 10-year period with a median age of 61 years (range 16–90 years). Among these MDS patients, the median OS was 36 months (range 1–125 months, 95% CI 24.02–47.98 months) and 28 patients (13.0%) progressed to AML. Basing on the 2016 WHO classification, all patients were classified as MDS as follows: 29 (13.4%) MDS-SLD, 60 (27.8%) MDS-MLD, 13 (6.0%) MDS-RS, 47 (21.8%) MDS-EB1, 48 (22.2%) MDS-EB2, 1 (0.5%) MDS-del(5q), 18 (8.3%) MDS-U. Besides, 185 patients were stratified into IPSS-R risk groups as follows: 14 (7.6%) very low, 41 (22.2%) low, 64 (34.6%) intermediate, 33 (17.8%) high and 33 (17.8%) as very high. Of these, the median IPSS-R score was 4.0(1.0–10.0) and the average score was 4.4. Further information was provided in Table 1.

**Table 1:** Characteristics of 216 patients with primary MDS

Variable	All patients	Elevated PMMBM group	Normal group	*P*-value
Age, years median (range)	61 (16–90)	62 (24–83)	61 (16–90)	0.971
Male/Female, n	117/99	23/13	94/86	0.200
BM blast, % median (range)	3.5 (0–19.5)	8.8 (0–19.5)	3.0 (0–19.5)	< 0.0001
PMMBM, % median (range)	3.0 (0–24.0)	7.5 (6.5–24.0)	3.0 (0–6.0)	< 0.0001
**Peripheral Blood**				
NE, × 10^9^/L median (range)	1.2 (0–7.4)	0.8 (0–6.6)	1.2 (0.1–7.4)	0.022
HB, g/dl median (range)	7.7 (2.2–14.2)	8.6 (2.9–13.4)	7.6 (2.2–14.2)	0.463
PLT, ×10^9^/L median (range)	51.5 (2.0–340.0)	42.0 (6.0–332.0)	55.0 (2.0–340.0)	0.143
Monocytes, ×10^9^/L median (range)	0.2 (0–0.8)	0.2 (0–0.7)	0.2 (0–0.8)	0.102
Monocytes, % median (range)	7.1 (0.4–39.0)	10.1 (0.7–27.2)	6.3 (0.4–39.0)	< 0.0001
SF, μg/L median (range)	304.4 (5.5–2612.0)	242.0 (9.5–1447.6)	307.0 (5.5–2612.0)	0.749
β2-MG, mg/L median (range)	1.8 (0–12.6)	2.2 (0.4–5.5)	1.8 (0–12.6)	0.058
LDH, IU/L median (range)	207.0 (54.0–1083.0)	215.0 (123.0–616.0)	203.5 (54.0–1083.0)	0.373
Cytogenetic abnormalities, % (n/n)	45.1 (83/184)	50.0 (15/30)	44.2 (68/154)	0.556
**WHO classification**				0.005
MDS-SLD, % (n/n)	13.4 (29/216)	8.3 (3/36)	14.4 (26/180)	
MDS-MLD, % (n/n)	27.8 (60/216)	14.0 (5/36)	30.6 (55/180)	
MDS-RS, % (n/n)	6.0 (13/216)	0 (0/36)	7.2 (13/180)	
MDS-EB1, % (n/n)	21.8 (47/216)	22.2 (8/36)	21.7 (39/180)	
MDS-EB2, % (n/n)	22.2 (48/216)	47.2 (17/36)	17.2 (31/180)	
MDS-del(5q), % (n/n)	0.5 (1/216)	0 (0/36)	0.6 (1/180)	
MDS-U, % (n/n)	8.3 (18/216)	8.3 (3/36)	8.3 (15/180)	
**IPSS-R cytogenetic** **risk**				0.756
Very good, % (n/n)	0.5 (1/185)	0 (0/30)	0.5 (1/155)	
Good, % (n/n)	66.5 (123/185)	56.7 (17/30)	66.5 (106/155)	
Intermediate, %(n/n)	20.0 (37/185)	26.7 (8/30)	20.0 (29/155)	
Poor, % (n/n)	2.7 (5/185)	3.3 (1/30)	2.7 (4/155)	
Very poor, % (n/n)	10.3 (19/185)	13.3 (4/30)	10.3 (15/155)	
**IPSS-R risk**				0.025
Very low, % (n/n)	7.6 (14/185)	0 (0/30)	9.0 (14/155)	
Low, % (n/n)	22.2 (41/185)	6.6 (2/30)	25.2 (39/155)	
Intermediate, % (n/n)	34.6 (64/185)	36.7 (11/30)	34.2 (53/155)	
High, % (n/n)	17.8 (33/185)	30.0 (9/30)	15.5 (24/155)	
Very high, % (n/n)	17.8 (33/185)	26.7 (8/30)	16.1 (25/155)	
Gene mutation, % (n/n)	68.8 (55/80)	77.8 (7/9)	67.6 (48/71)	0.811
Leukemia transformation, % (n/n)	13.0 (28/216)	22.2 (8/36)	11.1 (20/180)	0.070

Abbreviations: *BM* bone marrow, *PMMBM* the percentage of mature monocyte in bone marrow, *NE* neutrophil, *HB* hemoglobin, *PLT* platelet, *LDH* lactate dehydrogenase, *MDS-SLD* MDS with single lineage dysplasia, *MDS-MLD* MDS with multilineage dysplasia, *MDS-RS* MDS with ring sideroblasts, *MDS-EB* MDS with excess blasts, *MDS-U* unclassifiable, *IPSS-R* Revised International Prognostic Scoring System

### Elevated PMMBM in relation to clinical and laboratory factors

In our cohort, 216 patients were divided into two groups to analyze the correlation between elevated PMMBM and clinical and laboratory characteristics. It showed that the elevated PMMBM group had significantly higher counts of BM blast (*P* < 0.0001), higher PB monocyte percentage (*P* < 0.0001) and lower neutrophil counts (NE) (*P* = 0.022) as well as higher risk distribution in terms of IPSS-R (*P* = 0.025) compared with the normal PMMBM group. Also, the WHO subtype between these two groups had a significant difference (*P* = 0.005). Furthermore, in the elevated PMMBM group, 2 MDS patients were observed to evolve into CMML. There were no significant differences in other factors between two groups (Table 1).

### Elevated PMMBM was accompanied with more mutation of IDH2

**Fig. 1:**
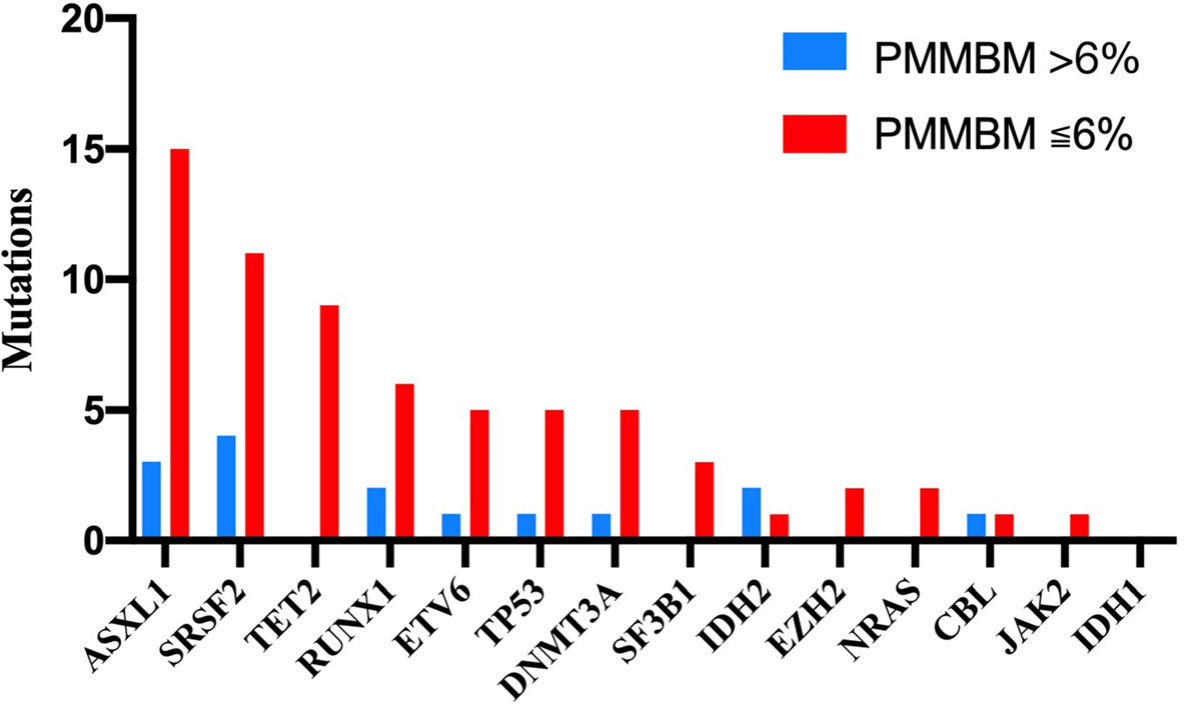
Type and frequency of 14 common gene mutations occurring in MDS patients including NRAS, DNMT3A, SF3B1, IDH1, IDH2, TET2, EZH2, JAK2, CBL, ETV6, TP53, SRSF2, ASXL1 and RUNX1

Mutations of 14 genes were detected in 57 patients, 41 (71.9%) of whom harbored mutations. Nine mutations with a minimum 5% frequency were identified in 14 genes in which ASXL1 mutation appeared the most (31.6%), followed by SRSF2 mutation (26.3%), TET2 mutation (15.8%), RUNX1 mutation (14.0%), ETV6, TP53 and DNMT3A mutations (both 10.5%), SF3B1 and IDH2 mutations (5.3%) (Fig. 1). The elevated PMMBM group harbored higher ratio of gene mutation in comparison with the normal PMMBM group, but the difference was not statistically significant (87.5% vs. 69.4%, *P* = 0.290). Among these mutations, the elevated PMMBM group showed higher mutation frequency of IDH2 compared with the normal PMMBM group (25.0% vs. 2.0%, *P* = 0.007).

### Elevated PMMBM was sociated with a poor prognosis

**Fig. 2:**
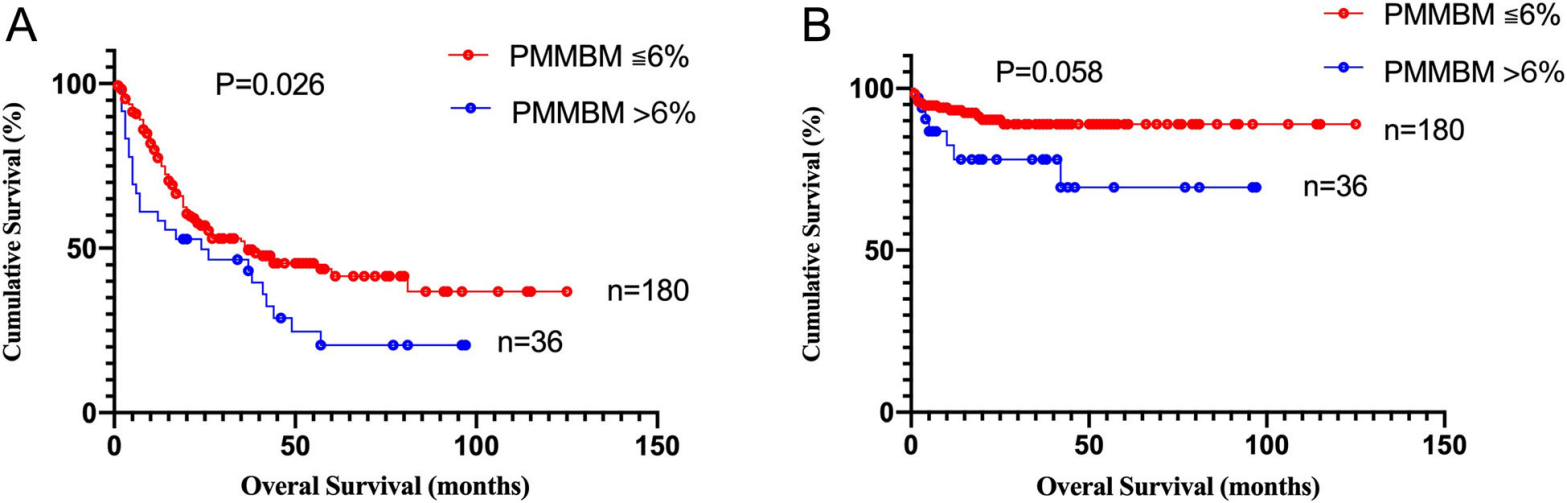
Overall survival and leukemia-free survival according to PMMBM in MDS. **a** Overall survival of 216 patients with primary MDS stratified by PMMBM≤6% vs PMMBM> 6% (*P* = 0.026). **b** Leukemia-free survival of 216 patients with primary MDS stratified by PMMBM≤6% vs PMMBM> 6% (*P* = 0.058)

Compared with the normal PMMBM group, the median OS in the elevated PMMBM group was significantly shorter (24 months vs 37 months, *P* = 0.026; Fig. 2a). But when it comes to LFS, the significance was in borderline (*P* = 0.058; Fig. 2b).

In univariate analysis, OS was adversely associated with older age (> 60 years) (*P* < 0.0001), male (*P* = 0.002), higher-risk IPSS-R (*P* < 0.0001), higher BM blast percentage (> 5%) (*P* < 0.0001), lower hemoglobin (HB) (< 10 g/dl) (*P* = 0.003), NE (< 0.8 × 10^9^/L) (*P* = 0.005) and PB monocyte counts (< 0.1 × 10^9^/L) (*P* = 0.012) (Table 2).

**Table 2:** Univariate and multivariate analyses for overall survival and leukemia-free survival in 216 patients with primary MDS

Variables	Univariate analysis for OS *P*-value	Multivariate analysis for OS *P*-value	Univariate analysis for LFS *P*-value	Multivariate analysis for LFS *P*-value
Age ≥ 60 (years)	< 0.0001	< 0.0001	0.438	–
Gender (male)	0.002	0.047	0.101	–
HB < 10 g/dl	0.003	0.080	0.554	–
NE < 0.8 × 10^9^/L	0.005	0.039	0.003	0.120
PLT < 100 × 10^9^/L	0.237	–	0.100	–
Monocyte < 0.1 × 10^9^/L	0.012	–	–	–
BM blast > 5%	< 0.0001	< 0.0001	< 0.0001	0.001
IPSS-R, cytogenetic risk group	< 0.0001	0.011	0.115	–
IPSS-R, risk category	< 0.0001	–	0.001	–
PMMBM≤6% vs PMMBM> 6%	0.026	< 0.0001	0.058	0.736

Abbreviations: *HB* hemoglobin, *NE* neutrophil, *PLT* platelet, *BM* bone marrow, *IPSS-R* Revised International Prognostic Scoring System, *PMMBM* the percentage of mature monocyte in bone marrow

Multivariate analyses showed that older age (> 60 years) (*P* < 0.0001), gender (male) (*P* = 0.047), higher BM blast percentage (> 5%) (*P* < 0.0001), lower NE counts (< 0.8 × 10^9^/L) (*P* = 0.039) and poor karyotype (*P* = 0.011) were adverse factors and elevated PMMBM was a significant prognostic factor for worse OS (*P* < 0.0001) but not for LFS (*P* = 0.736) (Table 2).

## Discussion

In our 216 MDS patients, elevated PMMBM was associated with a higher BM blast percentage at diagnosis, in accordance with higher IPSS-R scores. Our research suggested that elevated PMMBM was an independent adverse prognostic factor for OS.

A series of studies [14, 15, 21] have showed that a subgroup of MDS patients can evolve into CMML and present a poor prognosis. E. Schuler et al [22] conducted similar opinion that MDS patients with BM monocytic proliferation exerted CMML-like characteristics more often. However, the effect of elevated PMMBM on the prognosis of MDS remains unclear. To our knowledge, this study is the first to identify elevated PMMBM as the adverse prognostic impact on MDS. Recently, L Saeed et al [6] found that subnormal AMC of MDS was associated with an adverse OS in univariate analysis but not in multivariate analysis which is in accordance with our results. Nonetheless, elevated PMMBM is associated with adverse OS in our study.

The BM microenvironment is composed of BM stromal cells, mesenchymal stem cells, vascular endothelial cells, fibroblasts, mononuclear phagocyte system and cytokines [23]. Monocytes are essential cellular components of the host defense system. Due to their high plasticity, monocytes are involved in several cancer-associated processes including immune-tolerance, metastatic spread and neoangiogenesis along with M1 and M2-like macrophages induction [24, 25]. Recent studies have found that M2-like macrophages, called tumor-associated macrophages (TAMs), were involved in promoting tumor progression and metastasis by boosting angiogenesis, stimulating tumor cells’ proliferation, migration and invasion [26–28]. Although TAMs are initially considered to affect solid tumors, they are later found to predict poor outcomes in blood diseases such as lymphoma, leukemia and multiple myeloma [29]. But the roles of TAMs in MDS patients have not been fully elucidated. It is also considered that monocytes can contribute to tumor angiogenesis along with vascular endothelial growth factor (VEGF) to help tumor cells to evade the killing effect of immunocytes, and they can impede differentiation, maturation and proliferation of lymphocytes and promote survival of malignant T cells [30, 31]. Thus, we speculate that elevated PMMBM played an important role in the transformation and progression of MDS.

Further, it was demonstrated in our cohort that MDS patients with elevated PMMBM harbored higher BM blast percentage, PB monocyte count and especially IPSS-R score. It is well known that IPSS-R was widely used in measuring the prognosis of MDS since it was introduced in 2012 [5]. Our results showed that elevated PMMBM at the time of diagnosis significantly correlated with inferior outcomes and was closely accompanied with higher IPSS-R which is associated with a shorter OS.

In recent 10 years, recurrent somatic mutations in more than 50 genes have been demonstrated in 80–90% MDS [32], some of which are identified to predict the prognosis of this disease [7–10]. In our cohort, mutational analyses of 14 genes relevant to MDS were performed in 80 patients and elevated PMMBM patients harbored a higher mutational rate in IDH2.

IDH2 mutation as a DNA methylation regulatory gene can induce a block in cellular differentiation through epigenetic modifications, which plays an important role in contributing to premalignant disorders as well as oncogenesis [33–35]. It has been found in many solid tumors including gliomas, intrahepatic cholangiocarcinoma and enchondroma. Moreover, it has also rapidly been found in hematologic malignancies such as AML, MDS, myeloproliferative neoplasm, primary myelofibrosis and so on [36]. The prognostic impact of IDH2 in MDS remains controversial [8, 37–40]. But a large cohort study conducted that mutation of IDH2 was strongly associated with a short OS in MDS [41]. These results indicated that elevated PMMBM MDS patients could have distinct characteristics.

## Conclusions

In summary, we demonstrated that elevated PMMBM accompanied with higher frequency of IDH2 mutation was associated with a poor prognosis. PMMBM as a prognostic factor could assist IPSS-R to provide a convenient for measuring the prognosis of MDS patients especially when their karyotype analysis fails.

## Data Availability

The data that support the findings of this study are available from Ningbo First Hospital but restrictions apply to the availability of these data, which were used under license for the current study, and so are not publicly available. Data are however available from the authors upon reasonable request and with permission of Ningbo First Hospital. An Wu, the first author, should be contacted if someone wants to request the data from this study.

## Abbreviations

MDS: Myelodysplastic syndromes
PMMBM: The percentage of mature monocyte in bone marrow
OS: Overall survival
LFS: Leukemia-free survival
IPSS-R: Revised International Prognostic Scoring System
AML: Acute myeloid leukemia
IPSS: International Prognostic Scoring System
WHO: World Health Organization
WPSS: World Health Organization Classification-Based Prognostic Scoring System
MDAS: The MD Anderson Risk Model Score
CMML: Chronic myelomonocytic leukemia
BM: Bone marrow
PB: Peripheral blood
OM-CMML: Oligomonocytic CMML
AMC: Absolute monocyte count
HSCT: Hemopoietic stem cell transplantation
ISCN2016: International System for Human Cytogenetic Nomenclature (2016)
ROC: Receiver operating characteristic curve
NE: Neutrophil
HB: Hemoglobin
TAMs: Tumor-associated macrophages
VEGF: Vascular endothelial growth factor
PLT: Platelet
SF: Serum ferritin
β2-MG: β2-microglobulin
LDH: lactate dehydrogenase
MDS-SLD: MDS with single lineage dysplasia
MDS-MLD: MDS with multilineage dysplasia
MDS-RS: MDS with ring sideroblasts
MDS-EB: MDS with excess blasts
MDS-U: MDS with unclassifiable
